# Evaluation of a Proposal for Movement Integration in the Teaching–Learning Process in Early Childhood Education

**DOI:** 10.3390/children9020231

**Published:** 2022-02-09

**Authors:** Adriana Nielsen-Rodríguez, Ramón Romance, Juan Carlos Dobado-Castañeda, Francisco Javier Gil-Espinosa

**Affiliations:** Human Kinetics and Body Composition Laboratory, Department of Didactics of Languages, Arts and Sports, Faculty of Educational Sciences, Campus de Teatinos s/n, Universidad de Málaga, Andalucía Tech, 29010 Málaga, Spain; adriananielsen@uma.es (A.N.-R.); jcdobado@uma.es (J.C.D.-C.); javiergil@uma.es (F.J.G.-E.)

**Keywords:** early childhood education, physical activity, movement integration, active methodologies, active learning, accelerometry

## Abstract

Physical activity is essential to child development, but studies show that children are increasingly inactive. Due to schools being considered privileged environments to promote physical activity, the aim of this study was to increase the physical activity performed by early childhood education children during the school day by integrating movement into academic content and analyze this process. The amount and intensity of physical activity performed by a group of 24 3–4-year-old children in three different weeks were measured by accelerometry: one week with the methodology they had been following (week 1); and two weeks in which movement was integrated into the content through a specific proposal (week 2) and the same improved proposal (week 3). The results reveal that the application of a movement integration program not only allowed students to work on academic content in a physically active way, but also significantly increased the amount of physical activity that children performed during the school day. However, it was necessary to carry out several interventions the same day, or make movement integration the reference methodology, to meet the minimum recommended physical activity levels. In addition, to increase their effectiveness, interventions should be continually reviewed and improved to increase the motor engagement time.

## 1. Introduction

Studies conducted in recent years showed a worrying decrease in physical activity (PA) performed by young children [1,2]. In response to this problem, the World Health Organization (WHO) [2] recently published a document recommending that children aged 3 to 5 years should spend at least 180 min a day in PA, of which 60 min should be of moderate to vigorous intensity. In the same way, regarding the guidelines on time spent in sedentary activities, it indicates that children of these ages should not remain in this type of behavior for more than one hour at a time.

However, PA levels in children aged between 0 and 5 years are very low, and it is difficult to reach these minimum recommendations [3,4,5], since nowadays children are mainly engaged in sedentary activities during their free time, reaching between 39.49 min/h and 40.64 min/h [4,5].

With respect to schools, most pedagogical methodologies are too sedentary, with between 73% and 89% of the school day being dedicated to sedentary activities [5]. Furthermore, in the early childhood education stage, the performance of physical activities and specific motor skills sessions depends in most cases on the teachers, since the curriculum does not establish a schedule or a minimum time to be dedicated to psychomotor practice, and this results in a deficit of this type of session [6].

It is necessary to point out that adequate psychomotor work from the first years of life provides for better holistic and comprehensive development in children, while inactivity causes different disorders, diseases and difficulties in motor and cognitive development [3,5,7].

Therefore, the aim of this research was to increase the amount of PA performed by children in early childhood education during the school day by integrating movement into the academic content and analyze the characteristics of this process. The hypothesis was that integrating movement into the academic content in early childhood education would allow an increase in the PA that child population performed throughout the day, favoring an amount of exercise performed that was closer to what is proposed by official organizations.

### 1.1. Movement Integration through Play and Its Relationship with PA

One of the essential tools for psychomotor development in early childhood education is play, since it allows the acquisition of abilities, skills and knowledge, providing improvements in the physical, intellectual, affective, social, emotional and moral areas [8,9]. PA, for its part, causes physical, physiological and cognitive benefits. Therefore, both play and PA have a valuable impact on children’s health and development [10].

Play is a fundamental resource for imbuing academic content with movement, since it provides the motor activity that children need and is essential to the development of executive functions: it stimulates students’ motor and cognitive functions, ensuring that they are attentive to instructions and actively participate in educational tasks, achieving a significant improvement in students’ learning [8,10,11]. Furthermore, it involves a great effort to adapt to changes and overcome emerging problems [12]. All these cognitively demanding and novel demands that are presented in play can become learning experiences with an impact on the child’s real life.

Play invites children to get involved in order to enjoy, making them learn in a motivated and meaningful way. As described in self-determination theory, play generates intrinsic motivation, which is related to students’ success and well-being. At the same time, it generates the desire to continue learning, thanks to the situations and novel experiences that it provides and that children live becoming stimulating challenges that they have to overcome [13,14,15]. At the same time, it allows all the physical and cognitive aspects to be worked effectively and jointly and achieves a greater commitment and involvement of the students in the development of the educational tasks [8,16,17].

For all these reasons, motor play is defined as the best tool for learning in movement and as an exceptional and irrefutable resource in the overall growth of the individual [12]. The development of the physical, intellectual and social areas of children determines the nature of play, and this, in turn, helps the growth of the person in these three spheres. Therefore, if we want to understand, evaluate and promote physical activity in early childhood, it should be through play [8,9].

### 1.2. Movement Integration and Increase in PA in the Early Childhood Education School Day

Schools are an excellent environment to increase the time students spend in PA [4,11,18,19], mainly due to the valuable and numerous resources they have and the number of hours children spend in them.

One of the strategies to increase PA during the school day is the use of active methodologies, although it is possible to carry out many other and different strategies, as long as they are set up as part of an exhaustive PA program that enables and encourages children’s movement during their time at school [20].

Specifically, this study focused on the design and implementation of a program for integrating movement into academic content, that is, the introduction of PA at different intensity levels in class time oriented to the teaching of curricular contents [11,13]. This choice was due to the fact that these programs imply an improvement in students’ PA levels without having a negative impact on teaching time [3,4,11].

Nevertheless, schools find that several factors affect the creation and implementation of a movement integration program. The available space, the previous methodology used and the existence and characteristics of recess and psychomotor sessions, among other variables, have a direct impact on the levels of PA carried out by students [5,6,18].

However, there are many other elements to consider, such as a management team that supports the program, the characteristics and training of teachers, the limitations of the school context, the abilities of the students, their developmental stage, their differences and their preferences, as well as the time constraints that teachers face when planning and implementing new interventions, together with the workload they have to bear [4,5,11,19,20,21].

## 2. Materials and Methods

### 2.1. Recruitment, Participants, Ethics Approval and Consent to Participate

This research was carried out in a center that offers the second cycle of early childhood education (3 to 6 years old), in a class of the first year of this cycle. Within this framework, the class was chosen through convenience sampling, due to the impossibility of selecting specific individuals in the given classroom configuration. For this reason, this was a randomized cluster study, with children from the same school and classroom living in the same environment.

The sample was made up of 24 participants, 12 girls and 12 boys, aged between 3 and 4 years old (*M* = 3.5; *SD* = ±0.3). The study was approved by the Ethics Committee for Experimentation with Human Beings of the University of Malaga (code 114-2020-H) and was carried out under the Declaration of Helsinki [22]. All the procedures and interventions were reviewed and approved by the group teacher, with her collaboration at all times, and by the school principal, who filled out the respective collaboration agreements in writing, as well as the families. Likewise, written authorization was obtained from the parents (or guardians) before the children participated in the study, and after they had all received a document explaining and detailing the goals and methods of the research.

Participation in the study was voluntary and the confidentiality of the identity of the participants was always ensured. To do this, a unique identification number was used to anonymize all personal data, safely preserving all printed data in closed files and electronic information stored in a university laboratory computer protected by password.

### 2.2. Measures and Instruments

In the present study, we measured and analyzed the amount and intensity of PA performed by children between 3 and 4 years of age during the school day in three different weeks. In all the weeks, measurements took place during the normal class time while the academic contents were being worked on. In the first week, the normal methodology they had been following up to that moment was maintained (the pretest), but in the following two weeks, movement was integrated into contents through a specific proposal (week 2) and through the same improved proposal to increase the time of motor engagement (week 3).

Three instruments were used in this research: a stadiometer, a weighing scale and 24 accelerometers (one for each child). The first two instruments were used to obtain relevant information about the physical characteristics (height and weight) of the children who participated. To evaluate the amount and intensity of PA, we took into account the nature of childhood movement patterns, which are characterized by short bursts of intense activity that are interspersed with frequent episodes of lower intensity activity or rests [7,23]. Therefore, triaxial accelerometry was used as a consolidated method in the scientific literature, using the ActiGraph wGT3X-BT^®^ accelerometer (Actigraph, Pensacola, FL, USA), as it was considered the most valid, reliable and suitable for this purpose [24].

These accelerometers are characterized by their ability to measure acceleration differences, evaluating the magnitude and total volume of movement as a function of time. To do this, the electrical charge generated inside is filtered and converted into samples taken several times per second. These samples are summed over a previously specified period (epoch), and recorded in the internal memory of the accelerometer. After recording the magnitude of the accelerations during a given epoch in activity counts, the numerical integrator is reset and the process is repeated [23]. In this study, epochs were set at 1 s intervals to increase the accuracy of the variability of the infant activity collected by the accelerometer and obtain higher quality data [23].

Finally, we selected the cut-off points of Pate*,* et al. [25] to classify PA as sedentary (0–799 counts·min^−1^), light (800–1679 counts·min^−1^), moderate (1680–3367 counts·min^−1^) or vigorous (≥3368 counts·min^−1^).

### 2.3. Procedure and Data Collection

In this research, accelerometers were used to analyze PA patterns in the school context of a group of 3- and 4-year-old children during three consecutive weeks (one without intervention and two with intervention). The measurements were taken during school hours, placing the accelerometers upon the students’ arrival at school and removing them before they left. Although they can be placed on different parts of the body [23], on this occasion the accelerometers were fixed with an elastic belt on the iliac crest of the right hip of each participant.

Therefore, three different measurements were made, in which the accelerometers recorded the amount and intensity of PA performed within a movement integration program (two weeks) and outside of it (one week), in order to be able to compare the increase in the measured parameters.

First, an initial measurement was carried out as a pretest to evaluate the PA performed by the children and its intensity, as well as the time spent in sedentary activity during the school day for a week in which no intervention was carried out. Afterwards, an intervention based on motor play was implemented within the framework of a movement integration program. It should be noted that, for the selection of the activities included in this program, a review of the literature was carried out, consulting other programs that have been implemented and adapting different activities.

The intervention was carried out in two phases, each phase lasting one week. In the first phase, the activities initially designed for the movement integration program were implemented, which were later evaluated and improved in order to design and implement a second phase, in which it was intended to achieve a greater student motor engagement.

In general, one-and-a-half hour daily interventions were organized during the school day. During the interventions, the children participated in directed games through which they worked on the same contents that the teacher had planned. Depending on the needs, the activities were carried out inside the classroom, in the playground attached to the classroom or in a larger playground that was about 20 m away, and lasted between 10 and 20 min.

#### Description of the Activities

On the basis of a globalizing and holistic methodology suitable for early childhood education, physically active activities were designed to address the contents that had to be worked on in the classroom by the students. Some of these contents were the numbers 1 and 2 (spelling, quantity and counting), the seasons (objects, food and weather related), emotions and colors (yellow, green, red, blue, orange, purple, white, brown and black).

The activities designed were mainly based on games that involved transporting objects, displacements of different intensity and jumps, seeking the performance of motor-demanding actions. These activities could begin with the appearance of an object or a poster from which an explanation was created in the form of a story that introduced the activity, or through a prepared environment where the students entered and, without a need for explanations, the activity began.

The activities implemented were: “The lost treasure”; “The shopping list”; “The islands of numbers”; “An animal story”; “Together but not mixed”; “Every emotion in its own house”; “What’s the weather like?”; and “Color-color”.

In “The lost treasure”, children had to look for small colored psychomotor bags that had been hidden in different places and run to the chest of the corresponding color as quickly as possible to prevent someone from stealing them. In “The shopping list”, a supermarket was organized, with toys that represent food, and the students were told to go there to buy a quantity of food that was indicated either orally or by means of pictures with the spelling of the number.

In “The islands of numbers” two spaces were delimited, the island of 1 and the island of 2, and inside each one there were sheets with these numbers written in different sizes, shapes, colors etc., together with sheets of other numbers that were pirates. After explaining to the children that pirates have invaded the islands and that the 1 s and 2 s must be rescued, they had to move stealthily to the islands and pick up the cards with one of these two numbers on them, leave the others, and return to the starting point with long strides.

“An animal story” was a motor story in which various animals (a dog, a snake, a hummingbird, a crab, a kangaroo, an ant, a turtle etc.) experienced different emotions for different reasons, so that the children had to move around imitating the animal that appeared at that moment in the story and, when an emotion was named, they had to represent it. In “Together but not mixed”, the teacher played a variety of music and the children would dance until, suddenly, the teacher stopped the music and said a number. At that point, the students had to form groups of as many people as the teacher had said and stay in groups until the music started again.

In the activity “Every emotion in its own house”, areas or murals were placed that would be the houses of the different emotions (the house of joy, sorrow, anger, calm etc.), and pictures of people experiencing these emotions were distributed around the space so that the children had to move by jumping (with two feet, with one foot, with both feet together, forward, backward etc.) collecting the pictures and taking them to their corresponding house.

In “What’s the weather like?”, when the students arrived at the play area, they found objects, images and clothes of the four seasons of the year, so that they had to look for those that were related to the season that the teacher said (the name of the season or some characteristic could be said; for example, to take clothes from the coldest season). Finally, for “Color-color”, circles of different colors were distributed around the space and, when the teacher said a color, the students had to run to a circle of that color and stand next to it.

Once these activities were carried out and their results analyzed, they were modified to increase the motor engagement time using several strategies. On the one hand, the explanation time of the activities was reduced as much as possible to give more time for action. In addition, the spaces were reorganized to create more free areas that allowed a greater range of movements and displacements over longer distances.

On the other hand, the structures of the activities were repeated, changing their contents so that, as the students were familiar with the activity, they could perform it more autonomously and with greater confidence in their actions. However, at certain times the difficulty of the activities was increased, so that they would continue to be a challenge for the students and favor their motivation, always according to their possibilities.

For example, “The lost treasure” could start with a few colors and gradually add more, or chests could be placed separated from each other so that the children would have to go to different places depending on the chest they were looking for. In “Together but not mixed”, when the students were grouped according to the teacher’s instructions, instead of standing in place, they could be instructed to perform some action such as jumping, turning, crouching, etc. Finally, and in general, variations could be added in the movements and the displacements that the students would have to perform, as well as in the speed at which they would have to carry them out.

### 2.4. Descriptive and Statistical Analysis of the Data

Using the same computer in which the accelerometers were initialized, in order to avoid problems arising from possible desynchronization, ActiLife^®^ software (version 6.13.3; Actigraph, Pensacola, FL, USA) was applied to download and process the data. To prepare and clean the data, Microsoft Excel^®^ was used. Then, the filtered data were analyzed using the statistical package IBM SPSS^®^ 24.0 (IBM Corp, Armonk, NY, USA) in its Windows^®^ version.

Similar data processing criteria to those of similar studies was used [3,5,6,23]. Children’s physical activity levels were recorded during the school day, registering the total time spent in sedentary behavior, as well as their light, moderate, vigorous, and moderate to vigorous PA. It is necessary to point out that zero counts were not excluded in time-of-use calculations, because children can spend a lot of time sedentary in the classroom, and this was part of the data. To facilitate comparisons across weeks, all PA levels were averaged in minutes/hour.

In order for the data collected to be useful, only those accelerometers that were on or recorded data for a minimum of 3 h per day were considered (≥3 h of wear time) [7,23]. With all the data prepared, a descriptive and inferential analysis was performed, presenting the results as percentages, means and standard deviations. A significance level of *p* ≤ 0.05 was determined for the different tests.

Since the normality tests showed a non-normal distribution, non-parametric tests were applied for comparisons between weeks. To test if the amount and intensity of PA performed by the children in the different weeks were significantly different, the Kruskal–Wallis test was applied, followed by the Mann–Whitney U test (with Bonferroni correction) with the purpose of analyzing by pairs the differences in sedentary behavior and PA carried out in each week according to the implemented methodology.

## 3. Results

Table 1 shows the descriptive data of the sample regarding the age, weight and height of the children, indicating the means and standard deviations. In it, the differences between boys and girls groups are exposed, and it includes the data for the entire group.

The mean height of the sample was 99.1 ± 5.0 cm; the girls had a mean of 100.4 ± 4.2 cm, while the mean of the boys’ was 97.8 ± 5.5 cm. Regarding weight, the mean of the entire group was 16.9 ± 3.1 kg; the mean weight of the girls was 18.4 ± 3.5 kg, and that of the boys was 15.4 ± 1.8 kg. The mean age of the complete group was 3.5 ± 0.3 years.

Table 2 shows the descriptive values, in the form of mean and standard deviation, referring to the different levels of intensity of PA carried out by students during each week, expressed in minutes per hour. In addition, the same table shows the relationship between the total time of the school day and the minutes dedicated to PA, expressed as a percentage.

Regarding the PA performed, the analysis of the data revealed that children between 3 and 4 years of age with whom the study was conducted spent most of their class time sedentary (251.0 ± 10.3 min of a 300 min day, 83.7% of the total), the total PA took up the 16.3% of the school day (49.0 ± 10.3 min) and moderate to vigorous intensity PA was performed for only 10.4% of the school day (31.3 ± 7.5 min). However, when the movement integration interventions were carried out, differences were observed in the minutes spent on both sedentary and physical activities of different intensity.

With respect to the decrease in the time that students dedicated to sedentary activities, as well as the increase in total PA and in its different intensities, Table 3 shows the existing differences in relation to the different levels of intensity of PA according to each week.

Examination of both tables shows that the lowest levels of sedentary behaviors were recorded in week 3, with the second intervention (241.8 ± 13.3 min per day out of 300 total), although this number is still quite high (80.6% of the school day). This difference in time spent on sedentary behaviors is not as significant when compared to week 2 (first intervention) (*Z* = −0.770, *p* = 0.441). However, the difference from week 1 (*Z* = 2.407, *p* = 0.016), in which no specific intervention was carried out, is significant, that being the week with the highest values of sedentary behaviors (251.0 ± 10.3 min in a 300 min day).

Week 3 also had the highest level of light activity, which is again not significantly different compared to week 2 (*Z* = −0.242, *p* = 0.809) but it is significantly different when compared to week 1 (*Z* = −2.066, *p* = 0.039), as is the case of the time spent in sedentary activities. The same pattern is repeated if we refer to the amounts of vigorous, moderate to vigorous activity and light to vigorous activity: the highest levels of these intensities were found in week 3, the results not being significant compared to those of week 2 but significantly different from those of week 1.

The only difference was found when referring to moderately intense PA, which still reached its maximum in week 3 (19.7 ± 4.9 min), but does not represent a significant result when compared with week 1 (*Z* = −1.839, *p* = 0.66) or with week 2 (*Z* = −0.858, *p* = 0.391).

The levels of light PA were very similar in weeks 2 and 3 (20.2 ± 5.4 vs. 20.5 ± 5.7 min), and their differences with respect to week 1 are significant (*Z* = −1.979, *p* = 0.048; *Z* = −2.066, *p* = 0.039). However, this only occurs when referring to light PA, since when examining the results that refer to both sedentary time and the rest of the intensities of PA, no significant differences between weeks 1 and 2 were observed.

## 4. Discussion

The aim of our research was to increase the amount and intensity of PA performed by 3- and 4-year-old children during the school day through the use of a program for integrating movement into the curricular contents, that would contribute to reaching the levels of PA recommended by official organizations for this age range. This study also sought to analyze the characteristics of the movement integration process in order to improve interventions and help design an effective proposal to reduce sedentary lifestyles in early childhood education.

For this purpose, the amount of PA performed by the children during three weeks was measured and classified based on its intensity. In the first week, no intervention was performed, while in the second week a movement integration program was carried out, and in the third week the program was implemented again once it had been improved. Accelerometry data were collected in all weeks, thanks to which it was possible to compare the amount and intensity of PA performed during the school day depending on the methodology used.

The lack of interventions in this educational stage that combine PA and academic content in a way that is relevant to development and learning highlights the need for works such as this one. In fact, of the limited number of interventions found that truly integrate PA, based on movement to transmit or reinforce academic concepts, the vast majority have been carried out in primary schools, and very few have been carried out in early childhood education [3,4,11,12,16,17,24,26,27,28,29].

In general, and taking into account the cut-off points applied [25], our results indicate that during the week that no intervention was carried out the students spent most of the school day in sedentary activities (83.7% of the total time spent at school), dedicating 5.9% of the time to light-intensity activities, 5.6% to moderate-intensity activities and 4.8% to vigorous PA.

These results are similar to other analogous research that studied PA levels in preschoolers for one week while the children were in school. In those it has been shown that early childhood education children spend most of their school time in sedentary activities, their PA levels of any intensity being alarmingly low [3,5,6,7,19,21,23,24,30,31,32,33,34].

Moreover, it has been reported that there are variations in the amount and intensity of PA carried out depending on certain variables, such as, for example, the methodology employed, teaching styles, classroom organization, and opportunities to play that are offered in the school. In any case, it is essential to develop specific interventions, due to the fact that the PA that children perform at school is not enough to reach the recommended levels [5,7,18,21,35].

These data are alarming because they are far from the recommendations of international organizations, which insist that it is necessary for children under five to perform at least 180 min of PA per day. This PA can be of any type and intensity, although preferably a minimum of 60 of them should be aerobic and of moderate to vigorous intensity [2,36,37,38].

Along the same lines, with regard to the time dedicated to sedentary activities, it is specified that children of these ages should not remain immobile for more than 1 h at a time or sitting for prolonged periods. In addition, the time they spend passively in front of a screen should not exceed 1 h [2,39].

In terms of the type of activities, the most beneficial are those of moderate to vigorous intensity based on games, recreational practices, sports, displacement, physical education (school and/or extracurricular) or programmed exercises [2,8,40,41].

We understand that the school day is only part of the day, so it is not intended to reach the minimum recommended exclusively during school hours. However, taking into account that the school cannot address or know the PA that children perform at other times of the day, schools should try to approximate this minimum as much as possible in order to fulfill their responsibility to compensate for the possible deficiencies and difficulties that students may have to perform the necessary PA outside of school. Therefore, although the school cannot intervene in what happens outside of it, it does have the responsibility to change the current situation based on what happens inside.

It is true that covering the total activity time that children need only from school is complicated, but it seems to be possible to implement programs such as the one exposed in this research that help reduce the problem. We must remember that schools are very appropriate places to foster child PA, especially since children spend a large part of the day in them, which gives it a great scope and allows access to the majority of the child population on a regular basis [3,4,11,18,19,20,24]. However, it is very possible that this is not being carried out in schools, among other reasons, because current educational practices are mainly focused on the acquisition of academic content, primarily those related to arithmetic, reading and writing, thus decreasing the time dedicated to PA [3,4,9].

Therefore, it is essential to know what strategies can be used in schools and to recognize the opportunities for PA that exist in them to promote movement in children, especially by taking advantage of daily life in the classroom to incorporate PA into learning content [3,11,19,24,30,35]. In our study we presented one of them, which consists of the design and execution of a proposal for integrating movement into the teaching–learning process in which students can work on the curricular contents through activities based on motor games. This was supported by other similar research in which this type of strategy has also been used [3,4,11,16,19,24,30,32,41]. This strategy has several benefits, since in addition to increasing movement in school, it improves children’s cognitive activity and develops their executive functions, contributing to their increased academic performance [10,13,16,17,32,42] without affecting the teaching–learning process time [3,19,24,30,35].

Based on the data collected in Table 2 and Table 3, during a normal school day a child would reach, on average, 52.2% of the 60 min recommended in terms of moderate to vigorous intensity PA and 27.2% of the recommended 180 min of total PA. Given this, and according to other studies [4,6,19,24,30], class time corresponds to an excessively long sedentary period, which is significant over the whole day. For this reason, we should find a way for children to perform more daily PA, in this case from schools, since what is offered today is not enough [5,7,18,21]. 

To counteract this trend, a possible solution is the application of movement integration methodologies. The results of existing studies based on proposals to integrate movement into academic programs in early childhood education show that this practice considerably improves the PA levels of children during the school day without reducing the time dedicated to the educational process or the quality of teaching [3,4,12,17,24,35]. These results are congruent with our study, in which it can be seen that during the weeks of the intervention in which a movement intervention program was applied children’s sedentary lifestyle decreased (from 251 min/day in the non-intervention week to 241.8 min/day in the second intervention week). In addition, the amount and intensity of PA increased while working on the planned academic content in the teaching programming (from 49 min/day of total PA in the non-intervention week to 58.2 min/day in the second intervention week, and from 31.3 min/day of moderate to vigorous PA in the non-intervention week to 37.8 min/day in the second intervention week).

However, the increase in the total PA in the first intervention was not significantly different with respect to the control week, although the increase in light-intensity PA was significant. After the first intervention and after evaluating these results, we realized the need to refine the activities that had been implemented with the intention of improving the intervention and obtaining better results. When making the appropriate changes and intervening in the second week, significant results were obtained, both in the total PA time and in the time dedicated to light and vigorous intensity activities, as well as in the time spent on sedentary behaviors. Nonetheless, it was not possible to obtain a significant improvement in the time spent on carrying out moderate-intensity PA. This makes sense when studying the characteristics of the activity in these ages, which correspond to patterns that alternate explosive activity with periods of calm [7,23].

Evaluating the results of this research, it can be observed that, although with a daily session of an hour and a half the amount of PA performed is not enough, there is the possibility of increasing the number of sessions or their duration. This could be done by introducing, for example, a session before recess and another after, which would double the intervention time and, therefore, the total amount of PA carried out. Other studies have reached the same conclusion, and agree that the ideal would be to schedule, at least, two moments a day for the implementation of movement integration activities [6,7,9].

Considering the above, we can state that, if we want to foster both the amount and intensity of the PA performed by children in early childhood education, it is necessary to introduce movement opportunities through physically active academic proposals, although further research is needed to delve into the optimal balance between movement integration and other activities.

### 4.1. Factors Influencing Movement Integration Programs—Didactic Implications

Increasingly, physically active methodologies that combine movement with curricular content are appearing as a promising way to promote PA and learning at the same time [17]. Nevertheless, these proposals should be enriched by adding a component of playful experiences, since those have been proven to increase their benefits in all aspects [40].

It is well known that playing is a fundamental aspect in early childhood development and learning, and the implementation of structured play sessions (especially if they follow a model of interspersed activities and are of short duration, such as those presented in our research) are recommended as favoring a significant increase in PA [6,43]. 

In addition to this, most studies find positive associations between increased PA at school and improved executive functions, cognitive skills, classroom engagement, participation and academic behavior and performance [9,17,19,20,24,26,28,40,42,44]. This is because these interventions promote goal-directed behavior in students through tasks that provoke novel behavioral patterns, rather than seeking associative learning based on automatic behaviors [12]. Additionally, the execution of this type of complex motor movements involves neural circuits associated with executive functions, producing a better academic performance [12].

Therefore, if we take into account that movement integration into academic contents through structured play benefits not only PA, but learning and academic performance to a greater extent than other more sedentary teaching strategies [12], we can affirm that these methodologies are one of the best strategies to increase PA in children while enhancing their cognitive development and learning [16,19].

However, to carry out any PA integration practice in educational centers, it is necessary to consider the various factors that influence its implementation, thus helping to ensure the long-term maintenance of movement integration and other practices that favor children’s movement, as well as the effectiveness of future interventions [6,21].

Research findings have shown that “traditional” inactive teaching methods are used due to a lack of teacher training in alternative methods, space and logistical constraints on large group sizes, small classrooms, poor available resources and a lack of institutional support [19,35]. Active learning methods also initially require planning and preparation, leaving teachers concerned about whether they have the time, resources and energy to implement them [3,4,19,45].

In reference to teachers’ characteristics, the teachers of the school in which our study was conducted do not have any type of training in what refers to physically active methodologies or movement integration programs. For that reason, we agree with studies that state that it would be necessary to provide more training in designing and delivering movement integration programs, as well as promote greater awareness of the negative consequences of sedentary lifestyles [5,31]. This would contribute to the gradual replacement of sedentary lessons with more active ones [5]. Other influencing factors are the limited time available to teachers and the obligation to comply with the objectives of the curriculum, as well as having the support of the management team [4,19,20].

Student characteristics also influence the success or failure of movement integration programs [11,19,20], mainly because of their immature psychosocial and cognitive abilities [46]. At these ages, students’ attention span is short, they show a lack of concentration and distractions are frequent [10]. Children can act impetuously and spontaneously, are easily distracted, have difficulty waiting or sitting still for long periods and show poor perseverance in tasks [7,17,41,43,46].

For this reason, and in accordance with other similar investigations [7,11,19,20], in our proposal for movement integration the different activities designed involved brief explanations and did not extend too long in time. In addition, using play as a vehicle for learning is congruent with respect for individual growth and maturation rhythms [8,13]. Furthermore, we have verified that teachers should program spaces, materials and time to ensure that they are flexible, positive and stimulating in order to provoke learning through attractive and interesting proposals in which play, experimentation and practice are the main engine of the teaching-learning process, as was already evident in other previous works [8,13].

Movement integration programs can be used in both the classroom and outdoors. If outdoor spaces are used, as in our intervention, they should not be too far away from the classroom to avoid displacements that could end up affecting the motor engagement time. If a program is carried out inside the classroom, its configuration should be taken into account, since an adequate distribution of furniture and material, as well as a suitable free space, favor the proper development of the program. In any case, movement integration programs are characterized by providing a rich pedagogical environment that enhances engagement and concentration, and increases PA, relationships and motivation [11,13].

We must also consider a good temporal organization, since several studies, in the same way that ours has done now, have shown that a lot of time is lost due to the need to organize the material, explain the activities, move between spaces, or collecting the material, among other reasons [6,10].

As we can see, this research has numerous didactic implications for teaching practice in early childhood education. Data reveal that, if we want to increase the amount and intensity of children’s PA, it is necessary to implement physically active methodologies that allow and encourage movement during the school day. More specifically, we have developed a proposal to work on curricular content through activities based on motor play that were tested, evaluated and improved to increase the motor engagement time as much as possible.

After our experience, and in agreement with other authors [8,13,18,35], we consider that to develop an academic learning exercise in a way that also promotes the regular practice of PA, it is necessary that the design and implementation of play-based movement integration programs, in which different contents and skills, previously planned by the teacher, could be actively worked on by children. It is also important that in these sessions the children have the guidance and accompaniment of the teacher, who is responsible for preparing the material and the environment to generate the maximum learning and the most movement possible.

The design of play-based movement integration sessions has been shown to increase students’ motivation, enjoyment and participation, while having a positive influence on their physical, cognitive and social development in early childhood [3,8,32]. Teachers’ role is fundamental to this process by creating adequate learning-through-play spaces ensuring that children participate in the activities in the most profitable way possible, mainly through the instructions and guidance they can provide [21,43].

In summary, teachers have to ensure that their proposals for movement integration are attractive to their students, so they should carefully plan the form and the content of the activities together with the space organization, the schedule and the materials so that they promote physically active, playful and significant learning. However, for them to do so, they need to be trained in new methodologies based on physically active classroom interventions and at the same time given time to develop new programs, adapt the classroom and restructure the materials, as well as the necessary resources to implement this type of interventions.

Given the above, we can affirm that movement integration programs are one of the most favorable forms of increasing students’ PA while facilitating the construction of knowledge, problem solving and the enhancement of diverse skills and abilities autonomously, which favors the achievement of academic objectives and the development of skills that are essential to the positive development of students [16,19]. All this is thanks to the opportunities movement integration offers for cognitively demanding movements; applying knowledge in different contexts; stimulating action, reflection and expression; developing less concrete and more coordinated thinking; and applying divergent and convergent thinking [12,26].

Research such as the present study could be relevant to educational policy and decision-makers in order to design new strategies not only to promote childhood cognitive development and, as a consequence, better academic performance, but also to improve their health [4,24]. The inclusion of this evidence in good practice guidelines in education could lead to counteract the current school trend of reduced PA, and active movement integration methodologies could be widely disseminated to provide an effective means of improving the PA levels of a large population of children, also contributing to their educational improvement.

### 4.2. Study Limitations

This research has several limitations that should be reflected on. On the one hand, the interactions of the teachers with the children were not taken into account. The results of our work could undergo changes due to the role of the teachers during the interventions, and also according to the classroom methodology used. In this regard, research on the different methodologies used at this stage and how they can affect the amount of movement and PA performed by the children is very interesting.

On the other hand, the use of accelerometers has brought multiple benefits, but also some disadvantages that must be taken into account. Accelerometers provide an objective measure of students’ PA and sedentary behavior, and their use has minimized information bias. However, accelerometers mainly measure displacements and accelerations, so they overestimate the intensity of some relatively low-intensity activities, such as swinging, while underestimating the intensity of others, such as climbing, since the latter is an intense activity but involves slow displacements and no accelerations. This disadvantage has also been pointed out in other works in which it was specified that the type of activity carried out influenced the measured activity levels [6,7,21,23].

## 5. Conclusions

This study was carried out to discover the real repercussions that the implementation of a movement integration program in the teaching of academic content can have on the amount and intensity of PA performed in the early childhood education stage. Taking into account the current scenario regarding childhood inactivity, as well as the lack of interventions aimed at reducing school sedentariness in early childhood, research such as this one is necessary to make movement integration programs known.

Specifically, this work shows that an intervention of applying a movement integration program one and a half hours daily can lead to a significant increase in PA during the school day. However, this increase would not be enough to reach the recommended minimums, so it is considered necessary to carry out several interventions on the same day, so that the application time is increased, or to directly convert movement integration into the reference methodology.

It has also been shown that, to increase their effectiveness, interventions should be reviewed and modified to increase the duration of motor engagement while working on the content, since when this occurs the improvement in the amount and the intensity of PA becomes even more significant. In this study, we observed that the first intervention only improved the amount of light-intensity PA, while, after reviewing, improving and applying the program in the second intervention, both the total amount of PA and those of light, vigorous and moderate to vigorous intense PA were positively affected.

However, if we want to increase children’s PA during the school day by introducing physically active methodological proposals, some steps must be taken to enhance their success. On the one hand, it is necessary to train teachers so that they can design and implement movement integration proposals and also provide them with the resources and time necessary to do so.

On the other hand, it would be convenient to prepare the spaces and materials in advance, so that time is not wasted placing and collecting them, as well as using spaces close to the classroom to avoid long displacements, and selecting activities and tasks that require brief explanations to avoid losing students’ motivation.

Thus, this research aimed to promote the use of more active pedagogies in schools, such as movement integration programs, offering teachers accessible and realistic methodological alternatives that fit their possibilities and favor the integral development of students, and ensuring the maintenance and effectiveness of present and future programs.

## Figures and Tables

**Table 1:** Descriptive data of the sample.

	Boys (*N* = 12)	Girls (*N* = 12)	Total (*N* = 24)
	*M SD*	*M SD*	*M SD*
Height	97.8 ± 5.5	100.4 ± 4.2	99.1 ± 5.0
Weight	15.4 ± 1.8	18.4 ± 3.5	16.9 ± 3.1
Age	3.5 ± 0.3	3.5 ± 0.3	3.5 ± 0.3

Note 1: *N* = total number of the sample; *M* = mean; *SD* = standard deviation. Note 2: measurement units are height (cm), weight (kg) and age (years).

**Table 2:** Daily physical activity in minutes and percentages performed by students during the three weeks, and the overall mean for the three weeks.

	Week 1 (Pretest) (*N* = 23)			Week 2 (1st Intervention) (*N* = 24)			Week 3 (2nd Intervention) (*N* = 22)			Overall (*N* = 24)		
	*M*	*SD*	*%*	*M*	*SD*	*%*	*M*	*SD*	*%*	*M*	*SD*	*%*
Sedentary	251.0 ± 10.3		83.7	244.6 ± 13.0		81.5	241.8 ± 13.3		80.6	245.8 ± 12.7		81.9
Light	17.8 ± 3.5		5.9	20.2 ± 5.4		6.7	20.5 ± 5.7		6.8	19.5 ± 5.0		6.5
Moderate	16.9 ± 3.8		5.6	18.7 ± 5.0		6.2	19.7 ± 4.9		6.6	18.5 ± 4.7		6.2
Vigorous	14.4 ± 4.1		4.8	16.5 ± 5.5		5.5	18.0 ± 6.0		6.0	16.3 ± 5.4		5.4
MVPA	31.3 ± 7.5		10.4	35.2 ± 9.3		11.7	37.8 ± 9.6		12.6	34.7 ± 9.1		11.6
LVPA	49.0 ± 10.3		16.3	55.4 ± 13.0		18.5	58.2 ± 13.3		19.4	54.2 ± 12.7		18.1

Note: *N* = total number of the sample; *M* = mean; *SD* = standard deviation; MVPA = moderate to vigorous physical activity; LVPA = light to vigorous physical activity.

**Table 3:** Differences found in the levels of physical activity depending on the week.

	Week			Sedentary	Light	Moderate	Vigorous	MVPA	LVPA
*N* = 23	1	2	*p*	0.093	0.048 *	0.194	0.242	0.154	0.093
			*Z*	−1.681	−1.979	−1.298	−1.171	−1.426	−1.681
		3	*p*	0.016 *	0.039 *	0.066	0.017 *	0.019 *	0.016 *
			*Z*	−2.407	−2.066	−1.839	−2.384	−2.339	−2.407
*N* =24	2	1	*p*	0.093	0.048*	0.194	0.242	0.154	0.093
			*Z*	−1.681	−1.979	−1.298	−1.171	−1.426	−1.681
		3	*p*	0.441	0.809	0.391	0.301	0.367	0.441
			*Z*	−0.770	−0.242	−0.858	−1.034	−0.902	−0.770
*N* = 22	3	1	*p*	0.016 *	0.039 *	0.066	0.017 *	0.019 *	0.016 *
			*Z*	−2.407	−2.066	−1.839	−2.384	−2.339	−2.407
		2	*p*	0.441	0.809	0.391	0.301	0.367	0.441
			*Z*	−0.770	−0.242	−0.858	−1.034	−0.902	−0.770

Note: *N* = total number of the sample; MVPA = moderate to vigorous physical activity; LVPA = light to vigorous physical activity. Note 2: Mann–Whitney U test to compare medians between weeks; * *p* ≤ 0.05.

## Data Availability

The individual data of the participants are not publicly available, as specified in the original approval by the Ethics Committee on research with human beings of the Universidad de Málaga, Spain (protocol code 114-2020-H; approved on 26 February 2021) and in the informed consent from the participants and their respective legal guardians.
